# Supplementary material for: Discrimination between the Two Closely Related Species of the Operational Group B. amyloliquefaciens Based on Whole-Cell Fatty Acid Profiling
Source: Microorganisms. 2022 Feb 11;10(2):418. doi: 10.3390/microorganisms10020418 (PMC8877761; doi:10.3390/microorganisms10020418)
Supplement: Supplementary file 1 [file microorganisms-10-00418-s001.zip › microorganisms-1560952-supplementary.pdf]

Supplementary Materials

Table S1: Reference *Bacillus* strains and their sequence data using in phylogeny construction

Strain number	GenBank accession number	length (bp)	positions (bp)	length (bp)	positions (bp)	Reference	Origin
		gyrA		*rpoB*			
B. amyloliquefaciens							
LL3	NC_017190.1	937	7107 to 8043	743	117089 to 117831	[1]	isolated from fermented food (Korean bibimbap), China
TA208	NC_017188.1	937	7107 to 8043	743	112280 to 113022	[2]	industrial production of guanosine and synthesis of ribavirin, China
XH7	CP002927.1	937	7107 to 8043	743	112278 to 113020	[3]	industrial production of purine nucleoside inosine, China
B. velezensis							
AP183	CP029296.1	937	7136 to 8072	744	116972 to 117715	[4]	isolated from a cotton plant rhizosphere, USA
AS43.3	CP003838.1	945	7099 to 8043	744	122589 to 123332	[5]	Not provided
At1	CP041145.1	948	7133 to 8080	743	122719 to 123461	[6]	isolated from *Arabidopsis thaliana* seedling from surface sterilized seed, Uppsala, Sweden

Strain number	GenBank accession number	length (bp)	positions (bp)	length (bp)	positions (bp)	Reference	Origin
		gyrA		*rpoB*			
BIM B-439D	CP032144.1	943	7097 to 8039	742	122917 to 123658	[7]	isolated from the sample of soddy, ash-grey soil obtained on the territory of the Minsk region, Vileyka, Belarus
BvL03	CP041192.1	948	6608 to 7555	742	129097 to 129838	[8]	isolated from the sediment samples of fish pond, Wangcheng, Changsha, China
LF01	CP058216.1	937	2094067 to 2095003	742	2209767 to 2210508	[9]	isolated from tilapia, Guangzhou, China
QST713	CP025079.1	937	7100 to 8036	741	116750 to 117490	[10]	isolated from the commercial product Serenade (Bayer), France
S141	AP018402.1	945	7100 to 8044	742	122957 to 123698	[11]	isolated from soybean (Glycine max) rhizosphere, Thailand
SGAir0473	CP027868.1	956	2217071 to 2218026	743	2333101 to 2333843	[12]	isolated from tropical air, Singapore

Strain number	GenBank accession number	length (bp)	positions (bp)	length (bp)	positions (bp)	Reference	Origin
		gyrA		*rpoB*			
SQR9	CP006890.1	937	7100 to 8036	742	117092 to 117833	[5]	isolated from the plant rhizosphere soil, China
WRN014	CP041361.1	948	6790 to 7737	742	122609 to 123350	[13]	isolated from soil of banana root in fields, Hainan, China
B. subtilis							
ATCC 6633	CP039755.1	2466	3779374 to 3781839	743	3894578 to 3895320	[14]	Japan

References

1. Choi, J.; Nam, J.; Seo, M.-H. Complete genome sequence of *Bacillus velezensis* NST6 and comparison with the species belonging to operational group B. *amyloliquefacien*s. *Genomics* **2021**, *113*, 380–386. doi: 10.1016/j.ygeno.2020.12.011.
2. Grady, E.N.; MacDonald, J.; Ho, M.T.; Weselowski, B.; McDowell, T.; Solomon, O.; Renaud, J.; Yuan, Z.C. Characterization and complete genome analysis of the surfactin-producing, plant-protecting bacterium *Bacillus velezensis* 9D-6. *BMC Microbiol.* **2019**, *19*:5, doi: 10.1186/s12866-018-1380-8.
3. Fukumoto, J. Studies on the production of bacterial amylase. I. Isolation of bacteria secreting potent amylases and their distribution. *J. Agric. Chem. Soc. Japan* **1943**, *19*, 487–503, doi: 10.1271/nogeikagaku1924.19.7_487.
4. Dunlap, C.A.; Kim, S.J.; Kwon, S.W.; Rooney, A.P. *Bacillus velezensis* is not a later heterotypic synonym of *Bacillus amyloliquefaciens*; *Bacillus methylotrophicus*, *Bacillus amyloliquefaciens* subsp. *plantarum* and '*Bacillus oryzicola*' are later heterotypic synonyms of *Bacillus velezensis* based on phylogenom. *Int. J. Syst. Evol. Microbiol.* **2016**, *66*, 1212–1217. doi: 10.1099/ijsem.0.000858.
5. De Carvalho, C.C.C.R.; Caramujo, M.J. The various roles of fatty acids. *Molecules* **2018**, *23*:2583. doi: 10.3390/molecules23102583.
6. Dunlap, C.A.; Kim, S.J.; Kwon, S.W.; Rooney, A.P. Phylogenomic analysis shows that *Bacillus amyloliquefaciens* subsp. *plantarum* is a later heterotypic synonym of *Bacillus methylotrophicus*. *Int. J. Syst. Evol. Microbiol.* **2015**, *65*, 2104–2109. doi: 10.1099/ijs.0.000226.
7. Berezhnaya, A.V.; Evdokimova, O.V.; Valentovich, L.N.; Sverchkova, N.V.; Titok, M.A.; Kolomiyets, E.I. Molecular genetic and functional analysis of the genome of bacteria *Bacillus velezensis* BIM B-439D. *Appl. Biochem. Microbiol.* **2019**, *55*, 386–396. doi: 10.1134/S0003683819040033.
8. Cao, L.; Pan, L.; Gong, L.; Yang, Y.; He, H.; Li, Y.; Peng, Y.; Li, D.; Yan, L.; Ding, X.; Hu, S.; Yu, Z.; Sun, Y.; Huang, W.; Hu, Y.; Yi, G.; Xia, L. Interaction of a novel *Bacillus velezensis* (BvL03) against *Aeromonas hydrophila in vitro* and *in vivo* in grass carp. *Appl. Microbiol. Biotechnol.* **2019**, *103*, 8987–8999. doi: 10.1007/s00253-019-10096-7.
9. Geng, W.; Cao, M.; Song, C.; Xie, H.; Liu, L.; Yang, C.; Feng, J.; Zhang, W.; Jin, Y.; Du, Y.; Wang, S. Complete genome sequence of *Bacillus amyloliquefaciens* LL3, which exhibits glutamic acid-independent production of poly-γ-glutamic acid. *J. Bacteriol.* **2011**, *193*, 3393–3394. doi: 10.1128/JB.05058-11.
10. Borriss, R.; Chen, X.H.; Rueckert, C.; Blom, J.; Becker, A.; Baumgarth, B.; Fan, B.; Pukall, R.; Schumann, P.; Spröer, C.; Junge, H.; Vater, J.; Pühler, A.; Klenk, H.P. Relationship of *Bacillus amyloliquefaciens* clades associated with strains DSM 7^T and FZB42^T: A proposal for *Bacillus amyloliquefaciens* subsp. *amyloliquefaciens* subsp. nov. and *Bacillus amyloliquefaciens* subsp. *plantarum* subsp. nov. based on complete genome sequence comparisons. *Int. J. Syst. Evol. Microbiol.* **2011**, *61*, 1786–1801. doi: 10.1099/ijs.0.023267-0.
11. Fan, B.; Blom, J.; Klenk, H.P.; Borriss, R. *Bacillus amyloliquefaciens*, *Bacillus velezensis*, and *Bacillus siamensis* form an "Operational Group *B. amyloliquefaciens*" within the *B. subtilis* species complex. *Front. Microbiol.* **2017**, *8*:22. doi: 10.3389/fmicb.2017.00022.

12. da Costa, M.S.; Albuquerque, L.; Nobre, M.F.; Wait, R. The identification of fatty acids in bacteria. *Meth. Microbiol.* **2011**, *38*, 183–196.
13. Fan, B.; Wang, C.; Song, X.; Ding, X.; Wu, L.; Wu, H.; Gao, X.; Borriss, R. *Bacillus velezensis* FZB42 in 2018: The Gram-positive model strain for plant growth promotion and biocontrol. *Front. Microbiol.* **2018**, *9*, 2491, doi: 10.3389/fmicb.2018.02491.
14. Diomandé, S.E.; Nguyen-The, C.; Guinebretière, M.H.; Broussolle, V.; Brillard, J. Role of fatty acids in *Bacillus* environmental adaptation. *Front. Microbiol.* **2015**, *6*:813. doi: 10.3389/fmicb.2015.00813.

Table S2. Parameters of the whole-cell FA features of *B. velezensis*

Name of FA	Count of samples	Mean of FA content (%)	SD	SD / Mean	Minimum FA content (%)	Maximum FA content (%)	Usage
9:0	3	0.06	0.23	4.15	0.00	1.17	no
10:0 iso	2	0.01	0.06	5.15	0.00	0.39	no
10:0	4	0.03	0.13	3.69	0.00	0.69	no
11:0 anteiso	1	0.02	0.14	6.93	0.00	0.99	no
12:0	45	0.48	0.23	0.48	0.00	1.17	yes
13:0 iso	47	0.89	0.22	0.25	0.00	1.44	yes
13:0	3	0.01	0.06	4.64	0.00	0.39	no
14:0 iso	47	1.18	0.58	0.50	0.00	2.31	yes
14:0	48	2.87	0.70	0.24	1.69	4.89	yes
15:0 iso	48	30.39	2.53	0.08	25.63	35.87	yes
15:0 anteiso	48	32.13	2.33	0.07	27.20	36.42	yes
16:1 w7c alcohol	10	0.04	0.09	2.11	0.00	0.34	no
16:0 iso	48	1.70	0.77	0.45	0.67	3.09	yes
16:1 w11c	48	1.65	0.42	0.25	1.12	2.70	yes
16:0	48	12.53	1.82	0.15	8.90	17.49	yes
15:0 2OH	1	0.00	0.03	6.93	0.00	0.20	no
17:1 iso w10c	45	0.85	0.47	0.55	0.00	2.03	yes
17:0 iso	48	8.52	0.96	0.11	6.37	10.47	yes
17:0 anteiso	48	5.50	0.85	0.16	3.91	7.54	yes
17:0	26	0.17	0.18	1.06	0.00	0.69	no
16:0 iso 3OH	1	0.00	0.03	6.93	0.00	0.20	no

Name of FA	Count of samples	Mean of FA content (%)	SD	SD / Mean	Minimum FA content (%)	Maximum FA content (%)	Usage
18:3 w6c (6,9,12)	5	0.03	0.08	3.08	0.00	0.32	no
18:1 w9c	10	0.08	0.20	2.68	0.00	0.99	no
18:0	48	0.60	0.14	0.24	0.36	1.06	yes
19:0 iso	4	0.02	0.07	3.76	0.00	0.37	no
19:0 anteiso	2	0.01	0.06	4.86	0.00	0.29	no
19:0 cyclo w8c	1	0.00	0.02	6.93	0.00	0.13	no
18:1 2OH	1	0.00	0.03	6.93	0.00	0.23	no
18:0 3OH	2	0.01	0.07	4.86	0.00	0.36	no
20:0 iso	1	0.00	0.02	6.93	0.00	0.13	no
20:0	3	0.02	0.10	4.27	0.00	0.54	no
PC1		-0.13	3.33				
PC2		-11.37	2.26				
PC3		32.48	1.10				
PC4		-4.76	0.89				

Table S3. Parameters of the whole-cell FA features of *B. amyloliquefaciens*

Name of FA	Count of samples	Mean of FA content (%)	SD	SD / Mean	Minimum FA content (%)	Maximum FA content (%)	Usage
9:0	1	0.01	0.07	7.07	0.00	0.49	no
10:0 iso	4	0.06	0.21	3.57	0.00	1.04	no
10:0	6	0.09	0.27	2.88	0.00	1.22	no
9:0 3OH	1	0.01	0.10	7.07	0.00	0.70	no
11:0 anteiso	21	0.24	0.33	1.37	0.00	1.17	no
11:0	2	0.01	0.07	4.96	0.00	0.39	no
12:0	50	0.54	0.17	0.31	0.25	1.14	yes
11:0 2OH	1	0.02	0.11	7.07	0.00	0.76	no

Name of FA	Count of samples	Mean of FA content (%)	SD	SD / Mean	Minimum FA content (%)	Maximum FA content (%)	Usage
11:0 3OH	1	0.01	0.05	7.07	0.00	0.33	no
13:0 iso	46	0.50	0.19	0.37	0.00	1.01	yes
13:0	1	0.01	0.05	7.07	0.00	0.37	no
12:0 iso 3OH	1	0.01	0.04	7.07	0.00	0.29	no
12:1 3OH	2	0.01	0.05	4.95	0.00	0.27	no
14:0 iso	50	1.44	0.11	0.08	1.20	1.84	yes
14:0	50	0.61	0.14	0.23	0.44	1.02	yes
15:1 iso F	2	0.02	0.08	5.00	0.00	0.45	no
15:0 iso	50	27.84	1.65	0.06	24.12	31.09	yes
15:0 anteiso	50	31.92	1.98	0.06	27.76	35.16	yes
16:1 w7c alcohol	26	0.16	0.17	1.04	0.00	0.48	no
16:0 iso	50	3.51	0.19	0.05	3.02	3.94	yes
16:1 w11c	50	1.09	0.28	0.25	0.70	1.98	yes
16:0	50	4.57	0.55	0.12	3.14	5.99	yes
15:0 iso 3OH	1	0.00	0.02	7.07	0.00	0.17	no
15:0 2OH	2	0.01	0.06	5.00	0.00	0.34	no
17:1 iso w10c	50	1.07	0.37	0.35	0.71	2.31	yes
17:0 iso	50	15.92	1.96	0.12	13.38	19.03	yes
17:0 anteiso	50	8.99	0.73	0.08	7.65	12.65	yes
17:0	24	0.22	0.25	1.14	0.00	0.75	no
18:0 iso	16	0.10	0.15	1.56	0.00	0.50	no
18:1 w9c	11	0.10	0.23	2.37	0.00	0.95	no
18:0	49	0.59	0.23	0.38	0.00	1.17	yes
17:0 iso 3OH	1	0.01	0.04	7.07	0.00	0.26	no
19:0 iso	15	0.08	0.13	1.58	0.00	0.37	no
19:0 anteiso	3	0.02	0.09	4.24	0.00	0.48	no
19:0 cyclo w8c	3	0.02	0.08	4.13	0.00	0.44	no

Name of FA	Count of samples	Mean of FA content (%)	SD	SD / Mean	Minimum FA content (%)	Maximum FA content (%)	Usage
18:1 2OH	1	0.00	0.03	7.07	0.00	0.21	no
18:0 2OH	1	0.01	0.04	7.07	0.00	0.30	no
20:1 w7c	1	0.00	0.03	7.07	0.00	0.23	no
PC1		20.79	2.82				--
PC2		15.08	1.50				--
PC3		23.92	0.98				--

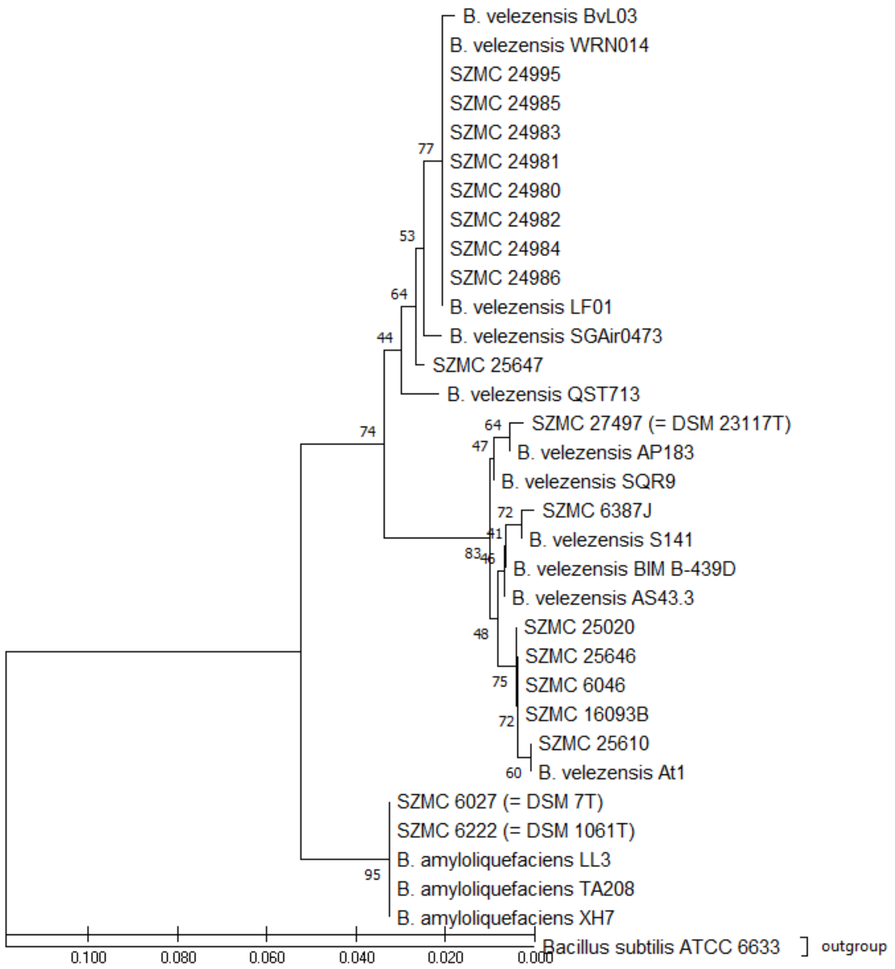

Figure S1. Neighbour-Joining phylogenetic tree based on *gyrA* gene sequences. Evolutionary distances were computed by the Tamura-Nei method. Bars, 0.020 substitutions per nucleotide position.

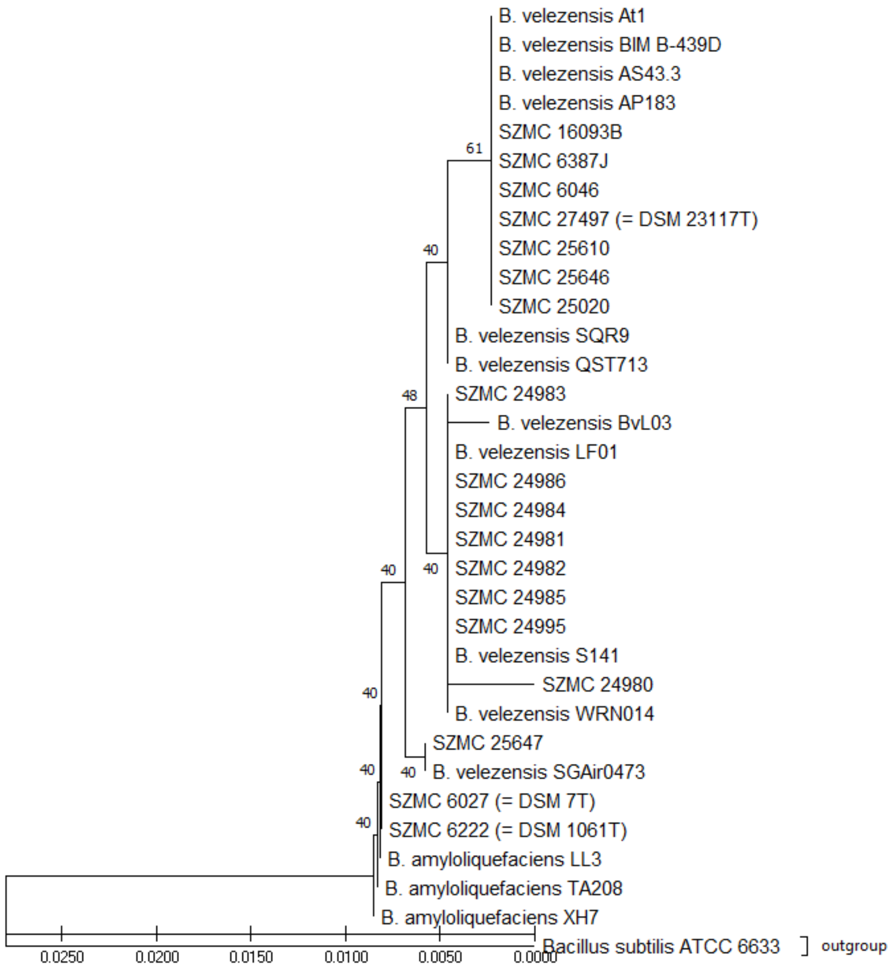

Figure S2. Neighbour-Joining phylogenetic tree based on *rpoB* gene sequences. Evolutionary distances were computed by the Tamura-Nei method. Bars, 0.0050 substitutions per nucleotide position.

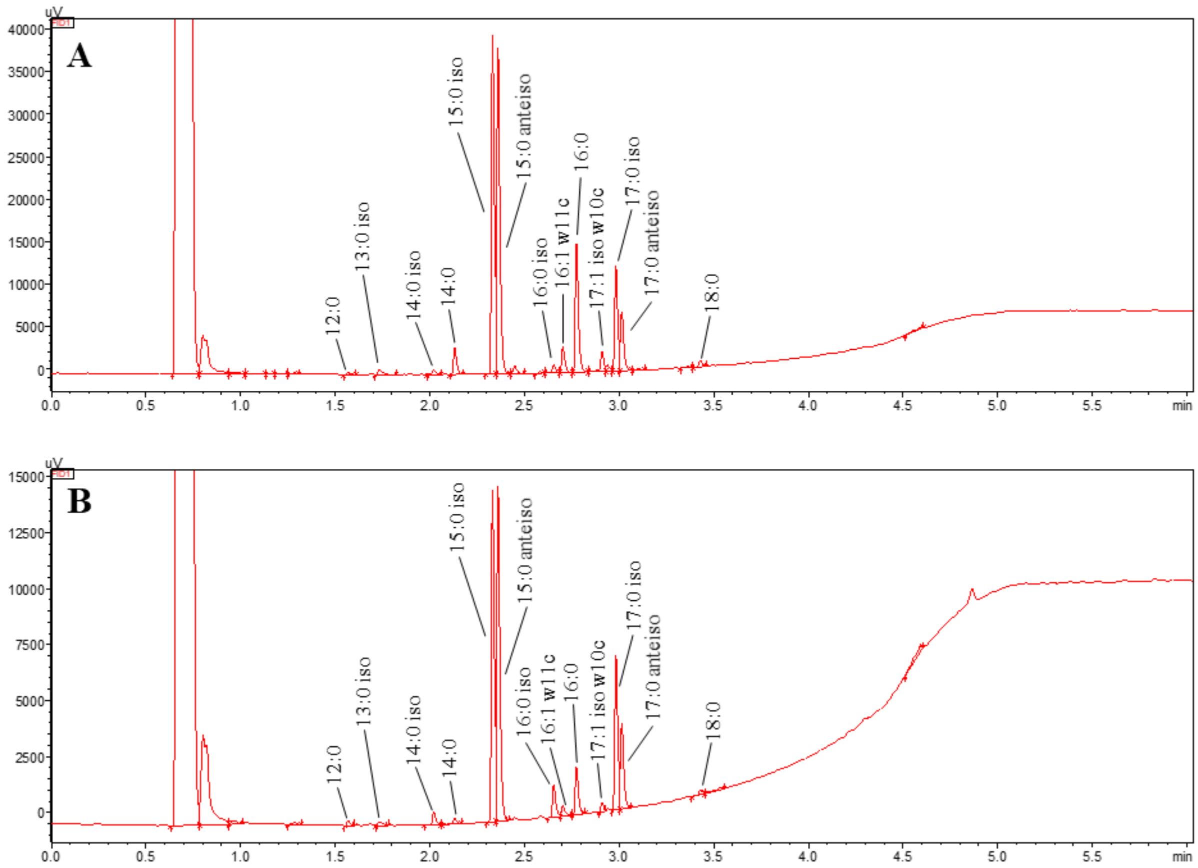

Figure S3. Chromatograms of the FAs of *B. velezensis* (**A**) and *B. amyloliquefaciens* (**B**).
